# Cardiometabolic Risk Factors in Women Exposed to High Altitude

**DOI:** 10.3390/ijms27146349

**Published:** 2026-07-17

**Authors:** Karen Flores, Karem Arriaza, Samia El Alam, Patricia Pérez, Eduardo Pena

**Affiliations:** High Altitude Medicine Research Center (CEIMA), Arturo Prat University, Iquique 1110939, Chile; kfloresu@unap.cl (K.F.); karriaza@unap.cl (K.A.); selalam@unap.cl (S.E.A.); paperezf@estudiantesunap.cl (P.P.)

**Keywords:** hypobaric hypoxia, women, cardiometabolic risk factors, altitude sickness

## Abstract

Currently, the number of people living and engaging in activities at high altitude is steadily increasing. Consequently, the physiological effects of altitude exposure have been extensively investigated to protect the health of individuals exposed to these extreme environmental conditions, alongside a notable increase in female participation driven by expanding opportunities in occupational, athletic, and residential settings. In addition to established risk factors, growing evidence suggests that cardiometabolic risk factors may increase the likelihood of developing altitude-related illnesses, while women present sex-specific physiological characteristics that contribute to metabolic disorders, including the menstrual cycle, contraceptive use, pregnancy, and menopause. Furthermore, factors such as hypobaric hypoxia, the type of activity performed at altitude, lifestyle, and emotional status may result in distinct physiological responses to altitude. However, little is known about the physiological adaptations experienced by women at high altitude, particularly regarding metabolic alterations that may contribute to impaired acclimatization. Therefore, this review synthesizes current evidence on the role of cardiometabolic risk factors and associated determinants in the development and exacerbation of altitude-related illnesses in women, aiming to identify existing knowledge gaps, guide future research, and improve prevention, acclimatization, clinical management, and women’s health and safety in high-altitude environments through a comprehensive assessment of current evidence.

## 1. Introduction

High-altitude activities are diverse and widespread across different regions of the world. These include sports, tourism, recreation, and occupational activities, all of which contribute substantially to economic growth and generate valuable employment opportunities [1]. In general, 0.74% of the global population, approximately 57.8 million people, reside above 2500 m.a.s.l (meters above sea level). High-altitude populations are concentrated mainly in the Andes, the Himalayas, and the Ethiopian Highlands [2]. Exposure to high altitude induces a state of hypobaric hypoxia due to the reduction in barometric pressure, which decreases the inspired partial pressure of oxygen and, consequently, oxygen availability to tissues [3]. Hypobaric hypoxia triggers a series of cardiovascular, neurological, and metabolic physiological responses that begin to manifest at approximately 2500 m and contribute to individual acclimatization to low-oxygen environments [4]. However, in some individuals, these adaptive responses may become maladaptive, leading to the development of altitude-related illnesses [5]. Therefore, research investigating the effects of hypobaric hypoxia has gained increasing relevance for the prevention and mitigation of altitude-related disorders, with the ultimate goal of improving health outcomes and quality of life.

In recent decades, women have shown increasing interest in exploring and engaging with high-altitude environments for various reasons. For example, female participation in demanding high-altitude occupational sectors in Chile has increased substantially, tripling over the past decade. By the end of 2025, women represented 23.1% of the workforce in these sectors [6]. Likewise, an increasing number of women participate in sports or live at high altitude. However, limited research has investigated the effects of altitude exposure in women. The physiological responses of women to high-altitude environments are particularly complex, as they involve a range of sex-specific factors that may influence adaptation to hypoxia. These factors include the menstrual cycle, hormonal contraceptive use, menopause, pregnancy, emotional status, metabolic disorders, and additional stressors associated with high-altitude exposure [7].

**Figure 1:**
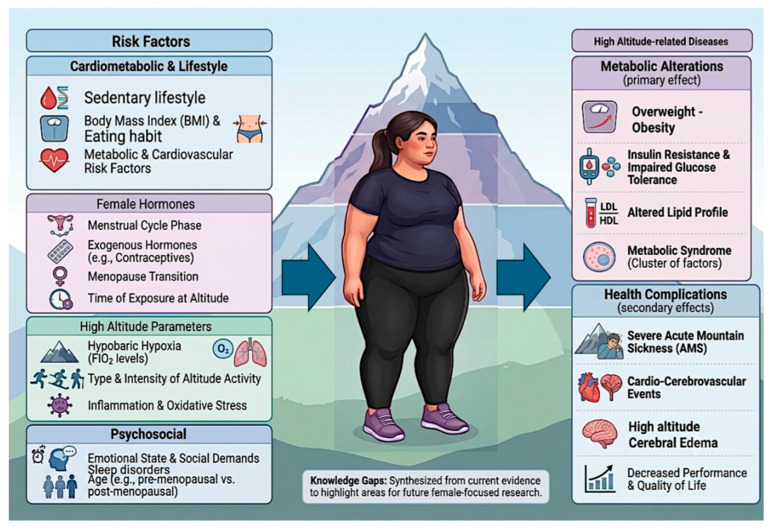
Conceptual framework illustrating the factors and metabolic outcomes influencing women’s health at high altitude generated by Gemini (Google AI, version 3).

Sex-based differences between women and men are well established and are primarily physiological in nature. These differences become more pronounced after puberty, particularly in relation to hormonal regulation and metabolic profiles. In general, women have lower muscle mass and a higher percentage of body fat compared with men, largely due to the influence of sex hormones such as estrogen. Estrogen plays a central role in energy homeostasis through its effects on cholesterol regulation, lipid metabolism, and adipose tissue distribution [8]. In addition, menopause and pregnancy are physiological states that may unmask pre-existing metabolic abnormalities and increase women’s cardiometabolic risk [9]. Cardiometabolic diseases are among the leading causes of death worldwide and represent a major global public health concern [10]. In Chile, cardiovascular diseases remain the leading cause of mortality, while overweight and obesity constitute major modifiable cardiometabolic risk factors. According to the most recent estimates from the Pan American Health Organization (PAHO) and the Food and Agriculture Organization (FAO), Chile has one of the highest prevalences of overweight and obesity in Latin America, with approximately 63% of adults presenting overweight and nearly 29–32% meeting criteria for obesity, with a higher prevalence among women than men [11,12]. Furthermore, elevated body mass index has been estimated to account for approximately 21,977 deaths annually in Chile, including more than 11,000 cardiovascular deaths, emphasizing the substantial contribution of obesity to the national burden of non-communicable diseases [13]. Despite this high burden, several studies suggest that chronic exposure to moderate or high altitude may induce metabolic adaptations including increased resting energy expenditure, enhanced glucose utilization and greater lipid mobilization that could partially attenuate cardiometabolic risk. Nevertheless, these adaptations are strongly influenced by altitude level, exposure pattern, lifestyle, ethnicity and nutritional habits, and therefore do not necessarily translate into lower obesity prevalence in all high-altitude populations [14,15] (Figure 1).

In this context, this literature review aims to analyze and synthesize current evidence on the influence of cardiometabolic risk factors and their associations with the development of high-altitude-related diseases in women who reside, participate in physical activity, or work intermittently at high altitude.

## 2. Women’s Physiology at High Altitude

Women exhibit sex-specific physiological characteristics that distinguish them from men, including the menstrual cycle phase, pregnancy, menopause, and hormone-related metabolic alterations, all of which may influence their physiological responses to altitude exposure. Additional factors, such as hormonal contraceptive use, should also be considered. However, studies investigating women at high altitude remain limited, inconsistent, and heterogeneous, partly due to substantial variability in hormonal fluctuations throughout the ovarian cycle [16,17]. Moreover, existing evidence has primarily focused on women who reside at or participate in sports activities at high altitude, whereas women engaged in high-altitude shift work remain significantly understudied. However, this knowledge gap is gradually being addressed, as the increasing participation of women in high-altitude occupational settings provides new opportunities for more robust and comprehensive research.

It is well established that above 2500 m, symptoms associated with hypobaric hypoxia begin to emerge as a consequence of the physiological challenges imposed by reduced oxygen availability and the need for acclimatization to extreme environments. However, when compensatory mechanisms are insufficient to maintain adequate adaptation, altitude-related illnesses may develop in susceptible individuals [18,19,20]. In hypobaric hypoxia conditions humans have limited ability to store oxygen in hemoglobin and myoglobin; consequently, exposure to low oxygen availability triggers a series of cardiovascular, hematological, neurological, and metabolic acclimatization processes [21]. In recent decades, considerable attention has been directed toward investigating sex-related differences in physiological responses to high-altitude exposure, particularly regarding susceptibility to and the development of major altitude-related illnesses, such as acute mountain sickness (AMS). AMS is characterized by headache, gastrointestinal disturbances, fatigue, weakness, and dizziness [4,22]. AMS in its most severe form, it can lead to high-altitude cerebral edema (HACE), which can be life-threatening and is marked by neurological signs such as ataxia, difficulty speaking, and decreased level of consciousness [23,24]. High-altitude pulmonary edema (HAPE), whose main pathophysiological mechanism is excessive hypoxic pulmonary vasoconstriction, together with the other two conditions, is caused by acute exposure to high altitude [25]. On the other hand, chronic mountain sickness (CMS) is characterized by excessive erythrocytosis, with pathological hemoglobin levels defined as Hb ≥ 19 g/dL in women and Hb ≥ 21 g/dL in men [26], and high-altitude pulmonary hypertension (HAPH); these latter conditions are caused by prolonged exposure at high altitudes [27,28]. It is important to mention that Andean high-altitude residents may also develop re-entry HAPE, a condition described particularly in residents returning to high altitude after spending time at low altitude [29].

Evidence suggests that women may exhibit a relative physiological advantage during high-altitude exposure, potentially mediated by higher endogenous progesterone levels, which may enhance ventilatory acclimatization to hypoxia [30]. There is also an advantage during pregnancy. A study showed that during pregnancy at altitudes of 3110 m and 4329 m, enhanced maternal ventilation and a greater ventilatory response to hypoxia improved arterial oxygen saturation, contributing to the maintenance of arterial oxygen content at levels comparable to those observed in nonpregnant women. These physiological adaptations were also positively associated with higher birth weight in the offspring [31]. Progesterone is recognized as a respiratory stimulant, and the modulatory role of estrogen is critical in enhancing this effect [32,33], which may contribute to improving oxygen saturation at altitude by increasing the hypoxic ventilatory response [34]. Based on the above, it has been reported that the incidence of HAPE may be lower in women than in men [25,35]. However, a higher incidence of HACE has also been reported in women exposed to high altitude (4300 m) [16], which may be related to increased cerebral blood flow associated with the effects of female hormones [36]. Moreover, regarding sex-related differences in AMS, current evidence remains inconclusive. However, a higher prevalence of AMS has been reported among women who are native to sea level following exposure to high altitude (>4000 m), where the phases of the menstrual cycle play a key role [17]. Finally, a study conducted among women living in the Himalayas at 4300 m reported a significantly higher prevalence of AMS compared with men [16]. These factors, among others, may contribute to the development of AMS in women, as discussed in this review.

A key finding highlighted in this review is that, following menopause, women exhibit a reduced ventilatory response to hypoxia, becoming comparable to that of men as progesterone levels decline. This physiological change may contribute to an increased susceptibility to altitude-related illnesses [37]. In addition, women exhibit distinct metabolic characteristics that may influence their physiological responses to high altitude, and factors that promote metabolic disorders may further increase this risk [38,39]. However, the available evidence remains inconsistent, and further research is needed to clarify the relationship between cardiometabolic risk factors and altitude-related illnesses in women.

## 3. Metabolic Disorders in Women at High Altitude

In metabolic terms, living at high altitude is associated with greater energy demand than those observed at sea level, along with alteration in plasma lipid and glucose concentration [40,41]. Reduced oxygen bioavailability promotes a metabolic shift toward greater glucose utilization and reduced free fatty acid uptake, as glucose is a more oxygen-efficient substrate for energy production than fatty acids [42]. Based on current evidence, an inverse association has been reported between altitude and the prevalence of metabolic syndrome, suggesting that chronic exposure to hypobaric hypoxia at high altitude may confer partial protection against metabolic diseases compared with residence at sea level [39]. However, one study showed a higher prevalence of metabolic syndrome in women in mountainous areas (>400 m) compared to sea level (52% vs. 33%) [43]. It is important to note that these differences may be influenced by several factors, including altitude, lifestyle, geographic location, dietary patterns, physical activity levels, ethnicity, and the duration and pattern of altitude exposure. Altitude exposure can be classified as chronic (individuals residing at high altitude), acute (individuals participating in short-term activities, such as sports or tourism), or intermittent (primarily associated with rotational or shift work at high altitude) [39,44,45,46]. However, most factors of metabolic syndrome are reversible, so keeping these disorders under control ensures a better stay in extreme conditions such as high altitude. Studies conducted in high-altitude populations (3825 m) have shown that lower resting oxyhemoglobin saturation is associated with increased cardiometabolic risk [47]. However, little is known about metabolic changes in people exposed to high-altitude shift work, considering factors intrinsic to this model, such as anxiety, eating habits, sleep disturbances, and lifestyle, and socioeconomic conditions, all of which may contribute to or exacerbate these metabolic alterations. It remains unclear whether these physiological changes are primarily driven by exposure to hypobaric hypoxia, the shift work schedule itself, or the combined effects of both factors [44]. A study demonstrated a significant association between shift work at sea level and a higher risk of metabolic syndrome in women compared with men [48]. Studies involving men engaged in high-altitude shift work and native high-altitude populations suggest that cardiometabolic risk factors may exacerbate the development of altitude-related illnesses, including AMS and HAPH [44,49]. Although the metabolic profile of women has been relatively well characterized under normal physiological conditions, their metabolic and physiological responses to high-altitude exposure remain poorly understood. Furthermore, the effect of the duration of high-altitude exposure on cardiometabolic risk in women has not been precisely determined [16]. However, cardiometabolic risk can be increased by factors related to the mode of exposure, such as shift work (involving intermittent hypobaric hypoxia) and the high-calorie diet characteristic and lifestyle sedentary of mining operations, which acts as a significant risk factor [44]. On the other hand, unlike women who live permanently at high altitude (chronic hypobaric hypoxia), where it has been established lower cardiometabolic risk [50]. Likewise, this has been observed among overweight/obese women performing 12 weeks of high-intensity interval training in high altitude who achieved greater reductions in body fat percentage and greater increases in lean mass and improved resting fat oxidation capacity [51]; this suggests that the duration of high-altitude exposure influences cardiometabolic risk factors in women.

Currently, in the general population, the prevalence of cardiometabolic risk factors is higher in women (21.7%) than in men (10%), according to the World Health Organization [10]. Similarly, in studies conducted at high altitudes, women living at high altitudes have a higher prevalence of metabolic syndrome (35.5%) than men (26.8%) [39]. Parallel outcomes were observed in women living at an altitude of 3900 m (women; 26.4% and men; 13.3%) [52]. In addition, menopause and pregnancy may unmask preexisting metabolic abnormalities and contribute to an increased cardiometabolic risk profile in women [9]. On the other hand, a study conducted in a Tibetan cohort reported a metabolic syndrome prevalence of 28.3% among women compared with 32.3% among men; these controversial results may be related to geographic locations and ethnic groups [53]. Likewise, women residing at altitudes above 3000 m in Derong, China, exhibited lower prevalence rates of metabolic syndrome [54]. Different body fat distribution patterns, particularly abdominal adiposity, adiponectin, and related biomarkers, may contribute to sex differences in cardiometabolic risk factors and to the prevalence of metabolic syndrome [55], which may potentially influence the prevalence and susceptibility to altitude-related illnesses in women.

### 3.1. Overweight/Obesity

Under hypoxic conditions at high altitude, overweight and obesity have been identified as factors that increase the risk of high-altitude illnesses, including AMS, elevated pulmonary arterial pressure, and right ventricular hypertrophy [44,56,57]. Hypoventilation is considered the primary underlying mechanism, as a higher body mass index (BMI) and greater waist circumference have been associated with impaired pulmonary function and lower arterial oxygen saturation SaO_2_ in individuals exposed to high altitude [35,58]. Waist circumference is one of the most significant factors associated with increased syndrome and cardiovascular risk. An elevated waist circumference (>102 cm in men and >88 cm in women) is considered a marker of central obesity and is strongly associated with adverse cardiometabolic outcomes [10]. At high altitude, increased visceral adiposity and overall body fat represent critical factors that may contribute to impaired ventilation and greater hypoxemia during exposure to hypobaric hypoxia. These alterations may promote the development of HAPH and potentially increase the risk of HAPE through mechanisms involving obesity hypoventilation syndrome and a heightened inflammatory state. In particular, elevated levels of interleukin-6 (IL-6) have been described in individuals with obesity, and this inflammatory pathway may also contribute to the pathophysiological mechanisms underlying HAPE [14,59,60].

Although previous studies have shown that overweight and obesity are inversely proportional to altitude, that is, their prevalence is lower at high altitudes [61,62], recent studies have revealed a high prevalence of weight-related disorders among residents living at high altitudes, particularly in women [63]. A study conducted in a town in Ecuador located at an altitude of over 1500 m found that the prevalence of obesity, low HDL cholesterol, and abdominal obesity was higher among women than among men [64]. Differences in the prevalence of obesity among women living at high altitude may be influenced by age, hormonal factors, lifestyle behaviors, including physical inactivity, and dietary preferences, ethnicity, and the duration of high-altitude exposure [55]. Altitude-dependent metabolic adaptations are not uniform but vary according to both the severity and duration of hypoxic exposure. Progressive reductions in oxygen availability induce coordinated changes in oxygen sensing, substrate utilization, energy homeostasis, and inflammatory signaling, resulting in distinct cardiometabolic responses across different altitude ranges. These adaptations include an increased reliance on glucose metabolism, progressive alterations in lipid utilization, changes in energy expenditure, and endocrine responses, particularly during prolonged or chronic intermittent hypoxic exposure [14,61]. Although most mechanistic evidence has been generated in mixed-sex populations, the available evidence in women suggests that hormonal status, body composition, and the characteristics of hypoxic exposure may further modulate these metabolic responses [15,65]. As summarized in Table 1, the available evidence indicates that cardiometabolic adaptations differ according to altitude range and the female population studied, including women undergoing acute ascent, permanent high-altitude residence, prolonged expeditions, and chronic intermittent hypobaric hypoxia associated with occupational exposure. However, studies directly comparing metabolic responses across different altitude levels in women remain scarce, highlighting an important knowledge gap for future research [15]. Importantly, the scarcity of studies directly comparing women across different altitude ranges limits our understanding of whether these metabolic adaptations are driven primarily by altitude per se or by differences in the duration and pattern of hypoxic exposure. A study of high-altitude residents in Peru found that women were more than three times as likely as men to have abdominal obesity (51% vs. 15%). The condition was also substantially more prevalent among women over 60 years of age, suggesting that both sex and age are important determinants of abdominal obesity [66]. Studies conducted among residents of Carhuamayo and Junín (4100 m) in Peru showed that BMI was significantly higher in women than in men (*p* < 0.01) [40]. Similarly, higher prevalence of abnormal waist circumference (64%) and obesity (14.1%) were found in women that living at high altitude [67]. Although sex differences in body fat distribution are well established, an abnormal accumulation of visceral adipose tissue remains a key factor in the development of obesity-related diseases. Consequently, sex-specific differences in body composition are expected to contribute to variations in the prevalence of obesity-associated metabolic and cardiovascular disorders. Women generally have a higher percentage of body fat and a lower proportion of lean mass than men, which may partly explain their greater susceptibility to abdominal obesity [8]. In addition, it is important to note that high altitude–induced weight loss is less pronounced in women than in men. In a study of 12 women who ascended to an altitude of 5050 m for 21 days, no significant changes in body weight or lean mass were observed compared with baseline values. These findings underscore the importance of investigating body weight and metabolic changes in women exposed to high altitude [68]. Information on women who perform shift work at high altitude is scarce, as most studies have been conducted in men and have shown a high prevalence of overweight and obesity among workers in the high-altitude mining industry exposed to chronic intermittent hypoxia [69,70]. Nevertheless, a study conducted among women working shifts at sea level reported that shift work was associated with a higher prevalence of overweight and obesity (62%) [45]. It is worth noting that, in Chile, high-altitude shift work is characterized by long workdays (approximately 12 consecutive hours), rotating day and night shifts, a sedentary lifestyle, nocturnal eating, and circadian rhythm disruption, all of which may contribute to weight gain and obesity [44,49,69,71]. Additionally, obese workers exposed to high altitude have been reported to be at increased risk of developing AMS [59]. Based on findings in men, it is therefore essential to study metabolic changes in women exposed to chronic intermittent hypobaric hypoxia.

### 3.2. Dyslipidemia

Dyslipidemia includes a range of abnormalities of lipid metabolism and may involve a combination of increased total cholesterol, low-density lipoprotein cholesterol, serum triglyceride, and lipoprotein levels or a decrease in high-density lipoprotein cholesterol, which are strong predictors of cardiovascular disease [72].

It has been well documented that exposure to high-altitude hypoxia is associated with dyslipidemia [73,74]. In general, studies about high altitude have found that dyslipidemia is more prevalent in women than in men. It has been reported that women living in Carhuamayo and Junín (4100 m) in Peru had higher levels of total cholesterol, LDL cholesterol, and low HDL cholesterol compared to men in the same location [40,67]. Similarly, a higher prevalence of hypercholesterolemia and triglycerides was observed in women (93.5%) than in men (50%) at 3600 m above sea level in Arequipa, Peru [75]. Another study of Tashkurgan Tajik Autonomous County (3100 m) indicates that serum ferritin levels are associated with metabolic syndrome and dyslipidemia mostly in women [76]. This is supported by studies that indicate that exposure to high altitude can alter the balance in lipid storage and mobilization in women and as a result generate a risk of chronic diseases caused by excess fat deposits and abdominal obesity [77], leading to overweight and obesity, which in turn contribute to the development of altitude-related diseases [60,78]. Scientific evidence has shown that at high altitude, plasma triglycerides increase through the overactivation of hepatic SCD-1, increased lipolysis in adipose tissue, increased secretion of hepatic lipoproteins such as VLDL, and decreased lipoprotein clearance [79,80,81,82]. Therefore, elevated triglyceride levels may represent another metabolic feature associated with high-altitude exposure. This finding is particularly concerning given the high prevalence of overweight and obesity among individuals exposed to high altitude, both of which are associated with increased plasma triglyceride concentrations. The concomitant changes in lipid and hormone profiles may reflect altered energy utilization in response to the negative energy balance experienced by women at high altitude. However, the mechanisms underlying these metabolic adaptations in women remain poorly understood and warrant further investigation. Furthermore, in male miners, night shift work has been associated with elevated total cholesterol, an effect that persists even among individuals reporting good sleep quality. These findings suggest that circadian misalignment contributes to lipid dysregulation through mechanisms that are independent of subjective sleep disruption [83].

### 3.3. Insulin Resistance/Hyperglucemia

It has been reported that the effects of high-altitude exposure on insulin depend on the duration of hypobaric hypoxia. Short-term exposure to hypobaric hypoxia has been consistently associated with increased insulin and blood glucose levels. For example, Larsen et al. reported a significant reduction in insulin sensitivity in a group of men after two days of exposure to a high altitude of 4550 m [84]. On the other hand, most studies in both humans and murine models indicate that chronic exposure to hypobaric hypoxia improves glucose metabolism by increasing insulin sensitivity [61,85]. Similar results are observed in animal models exposure to long-term intermittent hypobaric hypoxia, at different dose of hypoxia; studies with 4 h, 6 h, 8 h and 12 h per day of hypobaric hypoxia [86,87,88,89]. However, recent studies suggest that the development of insulin resistance depends more on the degree of tissue oxygenation than on the duration of exposure to hypobaric hypoxia. Accordingly, tissue hypoxia induces insulin resistance, which persists in individuals who are unable to achieve adequate tissue oxygenation. It has been observed that long-term high-altitude residents develop persistent insulin resistance when compensatory mechanisms fail to achieve adequate tissue oxygenation [90].

Insulin resistance develops following high-altitude exposure as an adaptive response and declines once compensatory mechanisms restore adequate oxygen delivery to the tissues. In individuals who are intolerant to hypobaric hypoxia, such as those who develop mountain sickness, persistent tissue hypoxia maintains insulin resistance and may predispose them to insulin resistance–associated complications, including essential hypertension, hypertriglyceridemia, reduced HDL cholesterol, visceral obesity, and non-alcoholic fatty liver disease [91]. As a result of rapid lifestyle changes associated with urbanization, high-altitude populations are experiencing an alarming increase in the prevalence of diabetes [92,93]. In addition, it has been reported that obesity- and high altitude–associated inflammation, together with activation of the sympathetic nervous system, contributes to glucose intolerance and insulin resistance [94,95]. Insulin resistance is widely recognized as an early pathogenic feature of conditions such as obesity and type 2 diabetes. Moreover, obesity is a major risk factor for the development of insulin resistance, and both conditions are well-established cardiovascular risk factors [96]. Also, it has been documented that insulin resistance has been implicated in the development of pulmonary hypertension [97]. A study of miners working at 4500 m in northern Chile on a 7-day-on/7-day-off shift schedule found that insulin resistance was associated with high-altitude pulmonary hypertension [44]. In addition, obesity-associated inflammation of perivascular adipose tissue may contribute to the development of vascular insulin resistance, thereby promoting hypertension [98]. Studies in women simulating a short-term exposure to an altitude of 4300 m for 16 h showed reduced insulin sensitivity, and carbohydrate utilization decreased [99]. On the other hand, a study in Quechua females living in rural villages in the Cuzco region (3400 m), showed a population average with the presence of insulin resistance [100]. Controversially, for women living in Cerro Pasco at 4750 m, the insulin sensitivity was higher at high altitude than women who live at sea level [101].

## 4. Hormones Alteration Associated with High Altitude

### 4.1. Adiponectin Alteration

Adiponectin is a hormone produced mainly by adipose tissue, known for beneficial cardiovascular and metabolic effects, enhancing insulin sensitivity, fatty acid oxidation, anti-inflammatory, anti-oxidant, and anti-atherogenic properties, promote vasodilation, regulate appetite and immune response [102,103]. In healthy individuals, adiponectin is abundant in the circulation. In obesity, adiponectin levels are decreased leading to an inverse association between metabolic disease and total plasma adiponectin [104,105]. This is because obesity is associated with elevated levels of the pro-inflammatory cytokine TNF-α, which contributes to chronic inflammation and suppresses adiponectin production [106]. Visceral fat accumulation is implicated in the dysregulated secretion of adipocytokines, which can contribute to the development of metabolic syndrome and cardiovascular diseases [107]. Consequently, low adiponectin levels are associated with increased insulin resistance and heightened inflammatory processes [106].

It has been well documented that in healthy subjects, adiponectin levels are higher in women compared with men [108]. This may be explained by the fact that lower adiponectin levels have been associated with increased visceral adiposity and greater waist circumference, conditions that are more prevalent in men than in women [109]. However, adiponectin levels have been closely associated with menopause, opening an important area for further investigation [54]. During the postmenopausal period, women experience an increase in visceral fat accumulation rather than preferential fat deposition in the hips, along with an increase in cardiovascular and metabolic risk factors, such as hypertension and dyslipidemia, due to the marked reduction in estrogen levels after menopause [110,111]. On the other hand, it has been observed that adiponectin levels tend to increase in people exposed to high altitudes (2900 m) [112]. Adiponectin levels increase during a 9-day exposure to high altitude (4750 m), possibly as a compensatory mechanism to meet increased energy demands in healthy mountaineers [113]. However, this response may be altered by preexisting metabolic conditions in individuals, such as metabolic syndrome, dyslipidemia, or other metabolic disorders characterized by low levels of adiponectin, which could disrupt the normal physiological response to altitude exposure [114]. Hypoxia activates inflammatory pathways in a manner similar to obesity [115]. Studies have shown that adiponectin levels decrease in response to increased inflammation and oxidative stress, along with reduced antioxidant capacity, which occur during exposure to hypobaric hypoxia at altitudes above 3000 m [116,117,118]. Additionally, AMS and adiponectin levels are linked through the body’s metabolic and inflammatory responses to hypoxia. Acute exposure to high altitude, particularly when associated with AMS, may reduce adiponectin levels, whereas prolonged exposure tends to increase adiponectin concentrations as part of the body’s adaptive response [112]. Ivana Gutwenger et al. reported that a 2-week structured training program at moderate altitude (1900 m) resulted in decreased adiponectin levels in individuals with metabolic syndrome [114]. It has also been reported that anorexia induced by high-altitude exposure suppresses adiponectin secretion. This reduction may contribute to the preservation of energy stores but could compromise processes involved in oxygen delivery and the utilization of oxygen-efficient fuels [119]. In general, little is known about adiponectin levels in women exposed to high altitude. Nevertheless, in women, these factors, combined with menopausal changes, may disrupt the regulation of adiponectin levels and increase the risk of developing conditions such as AMS. Interestingly, it has also been reported that exercise training under mild intermittent hypoxic conditions (2000 m) for 2 h per session, 4 days per week, over 8 weeks may more effectively reduce body fat and increase adiponectin levels in postmenopausal women compared with exercise training under normoxic conditions. These adaptations may contribute to a reduction in cardiovascular and metabolic risk factors [120]. The same finding was observed in a study with a shorter duration of hypoxia: mild physical exercise three times per week for 90 min in normobaric hypoxia for 8 weeks led to significantly greater weight loss in obese women [121].

### 4.2. Thyroid Hormone Alteration

The thyroid hormone plays a key role in regulating metabolism, growth, brain development, and protein synthesis by producing hormones such as triiodothyronine (T3) and thyroxine (T4) [122,123,124]. Thyroid disorders affect approximately 200–300 million people globally [125]. The hypothalamus–pituitary–thyroid (HPT) axis regulates hormone production; the pituitary gland releases thyroid-stimulating hormone (TSH), which stimulates the thyroid gland to produce T4 and T3. These hormones circulate in the blood and in peripheral tissues and are activated by deiodinases, which convert T4 into T3 [123]. Thyroid hormones are intimately related to the regulation of oxygen consumption at the cellular level; evidence from both human and animal studies suggests that the hypothalamic–pituitary–thyroid (HPT) axis is finely regulated to facilitate adaptation to hypobaric hypoxia conditions [126]. In addition to their well-established metabolic functions, thyroid hormones play an important role in adaptation to hypoxia by modulating erythrocyte function and facilitating oxygen delivery to tissues [127]. It has been documented that thyroid disorders are more prevalent in high-altitude regions than among populations living at lower altitudes. Factors associated with these disorders include environmental and lifestyle-related factors, such as exposure to air pollution, hypoxia, oxidative stress, intense physical exertion, psychological stress, sleep deprivation, cold exposure, and altitude-related changes in diet and gut microbiota, which may stimulate the thyroid axis and exert anabolic effects [122,128]. Previous studies have indicated that exposure to high altitudes above 3500 m increases thyroid hormone levels [119]. Nepal et al. compared thyroid function in Indigenous populations living at 2800 m and 3750 m and reported that the increase in free thyroid hormone levels at high altitude is not dependent on TSH secretion from the anterior pituitary [129]. Richalet et al. also conducted a study in men and observed that T3 and T4 levels increased progressively with increasing altitude. However, TSH levels released by the pituitary gland remained unchanged. This study concluded that the increase in thyroid hormone levels is not due to direct stimulation of the pituitary gland but may instead reflect adaptive mechanisms independent of pituitary regulation [130]. Nevertheless, in women living at sea level who participated in a high-altitude hiking expedition (>5000 m), a slight increase in TSH levels was observed [131]. Likewise, were evaluated eight healthy women participating in a mountaineering expedition in Nepal, here was observed that both thyroid hormones, TSH and T4, show an increase at altitudes above 4800 m [122]. In addition, it has been observed that among individuals living in a town located above 3000 m in China, women have a higher prevalence of hyperthyroidism than men living in the same area [132]. These findings support the notion that the female gonadal axis is highly sensitive to stressors and responds through dysregulation of hypothalamic–pituitary pulsatility [133]. It is important to note that pre-existing hyperthyroidism has been identified as a risk factor that may exacerbate high-altitude illnesses, such as HAPE and HACE, in women, indicating that thyrotoxic effects during high-altitude exposure may have potentially severe, even life-threatening, consequences [134]. The development of edema is exacerbated under these conditions because hyperthyroidism is associated with increased hyperventilation, vasoconstriction, and increased cardiac output, leading to increased cerebral and pulmonary blood flow, and consequently, higher capillary pressure in these tissues [135].

### 4.3. Involvement of Female Hormones

An important factor to consider regarding women’s responses to altitude is the influence of female sex hormones, particularly progesterone and estrogen. Several studies have shown that higher levels of progesterone and estrogen enhance the ventilatory response in women exposed to high altitude, thereby improving respiratory efficiency [136]. However, these responses decline with the onset of menopause, suggesting a role for hormonal modulation in the regulation of ventilatory responses [37]. The ventilatory response is a key adaptive mechanism to high-altitude exposure and has been proposed as a predictor of AMS, although findings remain controversial [137]. In this context, the luteal phase of the menstrual cycle has been proposed as a potentially advantageous period for women exposed to high altitude, given that elevated progesterone levels during this phase may enhance respiratory function [17,138]. Studies comparing different phases of the menstrual cycle have demonstrated that women in the luteal phase exhibit increased hypoxic and hypercapnic ventilatory responses during altitude exposure [17,139,140] but did not find differences in the occurrence of AMS between cycle phases [17]. However, the enhanced ventilatory response may support the hypothesis that women have a lower prevalence of pulmonary hypertension compared with men [141]. Likewise, no relationship has been found between the influence of these hormones and the development of AMS [30]. In addition, a study of women living at sea level who were exposed to an altitude of 3375 m for two days at the Torino Refuge (Courmayeur, Italy) showed no differences in ventilatory response or peripheral oxygen saturation between menstrual cycle phases or between women with and without AMS [137]. In fact, susceptibility to AMS in relation to sex hormones in women remains unclear, partly because it cannot be measured so easily [142]. In addition, progesterone and estrogen also have a crucial implication in metabolic status at high altitude, as they regulate the use of fats and carbohydrates [136]. In this context, AMS can be aggravated by metabolic changes [47]. Some authors have reported that women are more susceptible to developing AMS than men [17,143]. This may be explained by the influence of metabolic adaptations rather than hormonal fluctuations alone. Estrogen enhances insulin sensitivity and mitochondrial efficiency, whereas progesterone increases energy expenditure and influences the utilization of metabolic substrates. In addition, during the luteal phase, when progesterone levels are elevated, glucose consumption may increase during high-altitude exposure [17], because of its catabolic effect [144]. It is important to note that, with the onset of menopause, this situation changes, as the decline in female sex hormones contributes to the development of cardiometabolic risk factors [111].

It is also relevant to note that the menstrual cycle may be affected by high-altitude exposure; stress associated with travel and altitude has been reported to alter normal menstrual patterns. Women report changes in the heaviness of their bleeding, as well as absent periods, heavy menstrual bleeding, and irregular cycles during stays at high altitudes [145,146], although reassuringly, these irregularities have not been reported to be a risk factor for altitude sickness [147].

## 5. Other Associated Factors

### 5.1. Use of Hormonal Contraceptives

There is considerable uncertainty among women regarding the safety and effectiveness of hormonal contraceptives during travel to or residence at high altitudes. Many reports indicate that most healthy women can safely use contraceptives when traveling to high altitudes, although they should be aware of potential risks and limitations. It has been reported that none of the forms of hormonal contraception reduce their effectiveness in preventing pregnancy. However, it has been observed that combined oral contraceptives (estrogen + progestin) increase the risk of developing AMS, but it is not entirely clear whether there are risks associated with progestin-only pills, injectables, implants, hormonal patches, vaginal rings, or intrauterine devices [148]. More recent studies have not identified an association between contraceptive use and AMS [17,30,149]. Richalet et al. have observed in women at sea level who were exposed for more than 3 days to an altitude of 4000 m that physiological responses to hypobaric hypoxia depend on the phase of the ovarian cycle and menopausal status, and not on oral contraception or hormone therapy. In premenopausal women, the ventilatory response was greater during the early/mid-luteal phase than during the early follicular phase, and women with AMS had a lower ventilatory response, suggesting that there is a lower risk of developing AMS during the early/mid-luteal phase [17]. Conversely, a small study of women who rapidly ascended to an altitude of 3200 m showed that those using hormonal contraceptives were more susceptible to AMS, and that acetazolamide was less effective in preventing AMS in this cohort [150]. On the other hand, the use of hormonal contraceptives has also been linked to an increased risk of thrombosis. It has been reported that all forms of contraception carry an associated risk of developing thrombosis at altitude, but with a higher risk associated with oral contraceptives [151,152]. Currently, it has been reported that oral and patch combined contraceptive with estrogen increased the risk of venous thromboembolism and arterial thrombosis [149].

Specifically, oral contraceptive use has been suggested as a potential risk factor for the development of obesity, particularly when used continuously for periods longer than two years [153]. Additionally, oral contraceptive use has been associated with adverse changes in several metabolic, cardiovascular, and inflammatory parameters, potentially increasing the future risk of cardiovascular and metabolic diseases. Although oral contraceptives have been modified over time to reduce these adverse effects, their use may still represent a risk factor [153,154]. Most oral contraceptives contain ethinylestradiol as the estrogenic component. Although the lower doses of ethinylestradiol used in combined oral contraceptives have been shown to improve safety profiles, their use remains associated with serious cardiovascular adverse effects, such as venous thromboembolism, particularly in women with additional risk factors. Furthermore, ethinylestradiol increases the synthesis of several hepatic proteins and influences lipid and carbohydrate metabolism [155]. Based on the above, contraceptives may be a risk factor for thrombosis and cardiometabolic disorders, thereby increasing women’s risk of developing high-altitude diseases.

### 5.2. Psycho-Emotional State

A highly relevant parameter to investigate is the mental health of women exposed to high altitudes, whether through residence, occupational activities, sports participation, or tourism. In each of these contexts, several factors may contribute to the development of mental health disorders, including anxiety, depression, panic attacks, insomnia, and psychological stress. Excessive workload, social isolation, feelings of loneliness in remote and sparsely populated areas, and, particularly, being separated from children and family members are among the main contributing factors. It has also been reported that mood can be altered during high-altitude exposure [156,157,158]. It has been reported that, at low altitudes, the prevalence of anxiety and depression among women is twice that observed in men; therefore, these conditions could potentially become more prevalent with exposure to high altitudes. In fact, after several studies, the angiogenic effects of exposure to high altitudes have been reported [159]. A growing body of scientific evidence supports the association between high-altitude exposure and adverse mental health outcomes. For example, a study conducted among women living at high altitude (3800 m) reported an increased risk of short- and long-term emotional and psychological consequences, including mood disturbances, greater psychological burden, a higher prevalence of depression, and increased suicide risk [160]. Another study showed that an increased risk of AMS was associated with a medical history of anxiety disorders and depression among women participating in mountaineering activities on Mount Everest [161]. The development of depression, anxiety, and sleep disorders represents another relevant aspect, as these conditions have been associated with biological alterations at endocrine and neurological levels during high-altitude exposure. Alterations in the tryptophan (Trp)–serotonin–melatonin axis contribute to changes in mood and sleep patterns following high-altitude exposure. Hypoxia and insomnia may reduce serotonin availability, thereby contributing to anxiety and depressive symptoms [159]. Tryptophan is an essential amino acid for producing serotonin, a neurotransmitter involved in well-being, mood, and sleep. Serotonin is converted into melatonin, a hormone that regulates the sleep–wake cycle and also influences appetite, gastrointestinal motility, sexual behavior, pain, and, certainly, thermoregulation [162]. Hormonal changes during the menstrual cycle in women exposed to high altitude exacerbate mood swings and anxiety-inducing effects related to changes in serotonin; low estrogenic activity may result in low serotonin availability [159]. In addition, prolonged exposure to rotating night shifts at high altitude, involving extended work periods of approximately 12 h, may contribute to cognitive fatigue and psychological stress among both women and men working under these conditions [7,163,164]. Additionally, women appear to be particularly affected by sleep disorders in high-altitude shift work settings, and these disorders represent a major source of psychological and physiological stress [7,159]. Another important aspect to consider in this specific occupational context is the psychosocial stress experienced by women working in male-dominated environments, including challenges related to gender roles and expectations [1,165]. Likewise, depression, anxiety, and the lifestyle patterns that develop in these shift work systems also contribute to poor eating habits and nutritional imbalances, leading to cardiometabolic risk factors and an inability to acclimate to high altitudes [7,44].

As mentioned previously, high-altitude exposure also affects thyroid hormone levels, which may further influence serotonin regulation, thereby affecting mood and thermoregulation in women [166]. In general, the average rate of serotonin synthesis in the brain at sea level is already 1.5 times lower in women than in men [159]. Exposure to high altitude alters the circadian rhythm of melatonin, which is often also related to energy metabolism [167]. Thus, the tryptophan (Trp)-serotonin-melatonin axis is a field strongly linked to sex hormones, inflammation, and energy metabolism in women [168]. Mental health disorders have become increasingly prevalent over time, driven in part by competitiveness, social pressures, and chronic stress. Therefore, assessing mood and depressive symptoms before high-altitude ascent represents an important consideration.

## 6. Clinical Implications

The physical and mental health of women must be a priority for government and public health, especially when they are exposed to extreme environmental conditions such as high altitude. Conducting a risk assessment before any stay at high altitudes, considering factors such as metabolic and hormonal disorders, perimenopause and postmenopause, and mental health, will contribute to protecting women’s health through personalized treatments and mitigation strategies. This will reduce the prevalence of altitude sickness and cardiometabolic disorders, thereby lowering accident rates, work absenteeism, and healthcare costs, and most importantly, improving the quality of life for women exposed to high altitude.

## 7. Conclusions

The interaction between high-altitude hypoxia, cardiometabolic risk factors, mental health, and the distinct physiological characteristics of women represents a significant and emerging challenge for women’s health in extreme environments. Although current evidence suggests a potential increased susceptibility to metabolic disturbances and altitude-related illnesses, the existing body of knowledge remains limited and fragmented. Advancing research through a sex- and gender-specific approach is essential to better elucidate the underlying mechanisms involved. This will facilitate the development of targeted preventive and therapeutic strategies, ultimately promoting safer acclimatization and reducing health risks in women exposed to high-altitude conditions.

## Figures and Tables

**Table 1:** Altitude-dependent cardiometabolic adaptations reported in women across different altitude exposures.

Altitude Range	Molecular Mechanisms Involved	Main Cardiometabolic Changes Reported in Women	Female Population Studied	Representative References
**1500–2500 m** *Moderate altitude*	Oxygen sensing: ↑ HIF-1α; mild activation of hypoxia-responsive pathways	Slight increase in resting energy expenditure, early shift toward glucose utilization, mild sympathetic activation, generally preserved metabolic homeostasis	Healthy women during acute hypobaric exposure and short-term acclimatization	Woolcott et al., 2015 [61]; Raberin et al., 2024 [15]
**2500–3500 m** *High altitude*	Glucose metabolism: ↑ GLUT1/GLUT4, ↑ glycolysis; Energy sensing: initial AMPK activation	Increased carbohydrate oxidation, reduced lipid oxidation, enhanced glucose uptake, beginning of negative energy balance during prolonged exposure	Women during acute ascent, exercise studies and early acclimatization	Holden et al., 1995 [42]; Woolcott et al., 2015 [61]; Raberin et al., 2024 [15]
**3500–4500 m** *Very high altitude*	Energy metabolism: ↑ AMPK, ↓ mTOR; Lipid metabolism: ↑ lipolysis; Mitochondrial adaptation: ↑ PGC-1α	Greater fat mobilization, progressive negative energy balance, body composition changes, increased metabolic demands and endocrine adaptations	Female high-altitude residents and women participating in prolonged expeditions	Ermolao et al., 2011 [68]; Kayser & Verges, 2013 [14]; Gonzales & Tapia, 2013 [40]
**3500–4500 m** *Chronic intermittent hypobaric hypoxia*	Inflammatory signaling: ↑ NF-κB, ↑ ROS, ↑ IL-6/TNF-α	Increased insulin resistance, dyslipidemia, endothelial dysfunction, oxidative stress and chronic low-grade inflammation, especially when associated with shift work and obesity	Women exposed to rotating mining shifts (CIHH)	Lang et al., 2025 [7]; Brito et al., 2018 [44]; Pizarro-Montaner et al., 2020 [49]
**>4500 m** *Prolonged expeditions or extreme altitude exposure*	Persistent HIF signaling, sustained AMPK activation, oxidative stress and cellular stress responses	Marked negative energy balance, appetite suppression, skeletal muscle loss, endocrine alterations, impaired recovery and increased cardiometabolic vulnerability	Women during high-altitude expeditions or long-term residence at extreme altitude	Ermolao et al., 2011 [68]; Andjelkovic et al., 2024 [65]

## Data Availability

No new data were created or analyzed in this study. Data sharing is not applicable to this article.
